# Anatomical and functional outcomes after bilateral sacrospinous colposuspension (BSC) for the treatment of female genital prolapse

**DOI:** 10.1186/s12894-023-01213-w

**Published:** 2023-03-29

**Authors:** Wael Hosni, Carl-Michael Schmidt, Peter Mallmann, Sebastian Ludwig

**Affiliations:** 1 Department of Obstetrics & Gynecology, Marienhospital Brühl, A teaching hospital of the University of Cologne, Cologne, Germany; 2 Department of Obstetrics & Gynecology, University hospital, Cologne, Germany

**Keywords:** Sacrospinous colposuspension, Pelvic organ prolapse, Pelvic floor, Vaginal mesh

## Abstract

**Background:**

Pelvic organ prolapse is a bothersome condition affecting many women at advanced age, but also frequently observed in young women with certain risk factors. Various surgical techniques have been developed with the aim of providing effective surgical treatment for apical prolapse. The vaginal bilateral sacrospinous colposuspension surgery (BSC) with ultralight mesh and utilization of the i- stich is a relatively new minimal invasive technique with very promising outcomes. The technique offers apical suspension, in the presence or absence of the uterus. The objective of this study is to evaluate the anatomical and functional outcomes of bilateral sacrospinous colposuspension with ultralight mesh in 30 Patients treated with the vaginal single incision standardized technique.

**Methods:**

In this retrospective study, 30 patients were treated by BSC for significant vaginal, uterovaginal or cervical prolapse. A simultaneous anterior colporrhaphy, posterior colporrhaphy or both were performed when indicated. Anatomical and functional outcomes were assessed 1 year postoperatively using the Pelvic Organ Prolapse Quantification system (POP-Q) and the standardised Prolapse Quality of Life (P-QOL) questionnair.

**Results:**

The POP-Q parameters were significantly improved at twelve months after surgery compared to baseline. The total score and all four subdomains of the P-QOL-questionnaire showed positive trends and improvement at twelve months after surgery when compared to preoperative values. All patients were asymptomatic and expressed high satisfaction one year after surgery. No intraoperative adverse events were recorded for all patients. Only minimal postoperative complications were recorded and they all resolved completely with conservative management.

**Conclusion:**

This study highlights the functional and anatomical outcomes of the minimally invasive vaginal bilateral sacrospinal colposuspension with ultralight mesh for the management of apical prolapse. The one year postoperative results of the proposed procedure reflect excellent outcomes with minimal complications. The data published here are very promising and warrant further investigations and more studies to evaluate the long-term outcomes of BSC in the surgical management of apical defects.

**Trial registration:**

The study protocol was approved by the Ethics Committee at the University Hospital of Cologne, Germany (Date of registration: 08.02.2022) (Registration number: 21-1494-retro) (retrospectively registered).

## Background

Pelvic organ prolapse (POP) is a bothersome condition affecting many women at advanced age [1], but also frequently observed in young women with certain risk factors. High parity is the strongest risk factor for POP [2], however, other factors like obesity [3], neurologic injury to the pelvic floor [4], connective tissue disorders [5] and previous hysterectomy [6] have also been implicated.

Decreased vaginal or uterine support is seen in more than 30% of women presenting for routine gynecological examination. A women’s lifetime risk of surgery for symptomatic POP is 12–19% [7].

Conservative treatment with vaginal pessaries, pelvic floor physical therapy and local estrogen application in postmenopausal women is considered the first line therapy. Surgery is generally reserved for patients with symptomatic POP who have at least stage 2 prolapse on examination when conservative treatment methods have failed or rejected by the patient.

Apical prolapse refers to the downward displacement of the vaginal apex, uterus or cervix. Symptoms of apical prolapse include palpable or visible tissue protrusion, foriegn body sensation, pelvic pain and heaviness, dyspareunia and obstructed intercourse. Patients with apical prolapse often also complain of altered bladder und bowel functions that include obstructed voiding, urinary retention or incontinence, obstructed defecation and fecal urgency or incontinence [8].

Apical support is of paramount importance for the stability of the pelvic floor. It is now clear that a good apical support at the time of surgical treatment of POP is benificial for reducing the long term risk of prolapse recurrence [9].

Various surgical techniques have been developed with the aim of providing effective surgical treatment for apical prolapse. Historically, abdominal techniques such as abdominal sacrocolpopexy and vaginal approaches such as the unilateral Amreich-Richter operation were implicated (10–11).

Drawbacks to the abdominal approach were the significant intraoperative blood loss, long hospital stay and the increased risk of postoperative ilieus [12].

The main disadvantage of the vaginal unilateral sacrospinous attachment was the high recurrence of prolapse in the anterior wall, presumably due to posterior deflection of the vaginal apex [13].

To minimize the above mentioned drawbacks, laparoscopic approaches were developed. This includes the laparoscopic sacrocolpopexy, laparoscopic pectopexy and the laparoscopic lateral suspension [14–16]. While these techniques are minimal invasive and associated with excellent outcomes, vaginal apical repair may still be preferable in terms of the shorter operative time, quicker recovery and avoidance of any abdominal incisions [17]. This is even more appealing when treating patients with a history of multiple abdominal surgeries, obese patients and patients with significant concomitant comorbid conditions.

Farnsworth proposed the intravaginal slingoplasty surgery for the management of vaginal vault prolapse [18]. The surgical technique was promising in a small series of cases, however, rectal injuries led to abandonment of the technique.

For patients who choose to avoid mesh, vaginal bilateral sacrospinal colposuspension with sutures as means of native tissue repair is another treatment option. The bilateral approach allows the vagina to lie in a more horizontal plane, lower the incidence of proximal vaginal narrowing, de novo dyspareunia and bowel dysfunction. However, recurrence and anterior compartment failure rates are still a limitation [19].

Kieback proposed a bilateral sacrospinous colposuspension technique (BSC) with ultralight mesh as a novel, minimal invasive, reproducible method for the treatment of apical prolapse [20]. The procedure utilizes a very light polypropylene mesh with very high porosity leading to rapid anatomical integration and minimal tissues reaction. The mesh is fixed vaginally to the sacrospinous ligaments bilaterally with the help of an i-stitch anchoring instrument.

The vaginal bilateral sacrospinous colposuspension surgery with BSC-mesh and utilization of the i- stich is a relatively new minimal invasive technique with very promising outcomes. The technique offers apical suspension, in the presence or absence of the uterus.

The aim of this study is to evaluate the anatomical and functional outcomes of the bilateral sacrospinous colposuspension with BSC-mesh in 30 patients treated with the single incision standardized technique.

## Methods

### Design and data collection

This retrospective, single center study involved 30 patients who underwent vaginal bilateral sacrospinous colposuspension surgery with the ultralight implant and the single incision technique at Marienhospital Brühl, a teaching hospital of the University of Cologne, Germany. All surgeries were performed between January 2018 and July 2020. The study protocol was approved by the Ethics Committee at the University Hospital of Cologne (Registration number: 21-1494-retro).

All patients included in the study had significant vaginal, uterovaginal or cervical prolapse and were treated with BSC according to the published single incision standardized technique. All patients receiving the surgery were presenting with symptomatic stage II prolapse or higher. We have only included patients that were able to show up for postoperative evaluation 12 months after the surgery.

Most patients had additionally either a cystocele, rectocele or combined cystorectocele. A simultaneous anterior colporrhaphy, post colporrhaphy or both were performed when indicated. A simultaneous hysterectomy was only performed in patients with an associated uterine pathology, for example symptomatic fibroids.

There was no simultaneous TVT or TOT placement at the time of surgery in patients complaining of stress urinary incontinence (SUI).

All patients were well counseled and informed consents were signed preopratively. Preoperative assessment and postoperative follow-up included a vaginal examination and a pelvic ultrasound. Patients were asked to answer the female pelvic floor questionnaire before, as well as 12 months after the surgery. All patients were examined according to the pelvic organ prolapse quantification system (POP-Q) during the preoperative assessment and 12 months after surgery.

The primary outcome measure was the improvement of the pelvic floor symptoms and the secondary outcome measure was the anatomical correction of prolapse. The following data were retrospectively collected, statistically analyzed and used to assess the functional and anatomical outcomes of the BSC technique:

- Patients characteristics: Age, BMI, parity, menopausal status and surgical history of hysterectomy.
- Perioperative and postoperative complications.
- POP-Q quantification: preoperative assessment and at least one year postoperative.
- Standardized quality of life questionnaire: preoperative assessment and at least one year postoperative.

The premanufactured BSC-Kit (A.M.I. Inc.) includes an ultralight polypropelene U-shaped mesh with two loading units of the i-stitch instrument was used for the surgery.

At a material weight of 21 g per square meter; the entire BSC-implant weighs around 0.085 g. The implant has a very high porosity of 93% and is isoelastic. These properties allow an elastic form of suspension for the uterus or vagina with a minimum of foriegn body.

### Surgical intervention

All surgeries were conducted in general anesthesia and performed by two experienced designated urogynecological surgeons. A perioperative single dose i.v. antibiotic prophylaxis with a combination of cephalosporin and metronidazole was administered. The patients were placed in lithotomy position. The vagina was thoroughly disinfected, bladder emptied with intermittent catheterisation and draping was achieved. All surgeries followed the standardized previously published 10 surgical steps.

1. Preoperative treatment: At least two weeks of vaginal estriol pretreatment for postmenopausal women was mandatory.
2. Vaginal wall incision: The incision site is infiltrated with a vasoconstringent medication. The site of the vaginal incision depended on whether a simultaneous anterior colporrhaphy or posterior colporrhaphy were performed. A 3–4 cm longitudinal incision is made in the midline, either on the anterior or posterior vaginal wall.
3. Bilateral access to the sacrospinous ligament: The access canals to the sacrospinous ligaments on both sides are prepared using minimal sharp dissection with fine scissors and blunt dissection with the index finger. There is no need for extensive tissue dissection or visualization of the sacrospinous ligaments.
4. Preparation of the vaginal apex or cervix: The dissection and mobilization of the cystocele or rectocele from the vaginal wall is performed at this point. The horizontal space under the vaginal apex is dissected and prepared for the subsequent attachment of the BSC-tape. In cases with intact uterus; the anterior or posterior walls of the cervix are prepared for the attachment of the tape.
5. Suture placement for fixation on the sacrospinous ligaments: The I-Stitch instrument guided with the surgeon’s index finger is introduced into the previously prepared canal. The index finger now pushes the tip of the I-Stitch into the tissue of the sacrospinous ligament at the desired point of fixation. The suture is advanced into the ligament and the instrument is retracted and removed. Traction is then applied to the suture to test its stability. Knotting is not made at this point. The suture is fixed on the thigh with adhesive tape. The same procedure is repeated on the other side.
6. Suture placement for central fixation of BSC-mesh: Two sutures are placed in the midline, either on the cervical wall or the vaginal apex. In cases where anterior approach is performed; the mesh is fixed on the anterior wall of the cervix. In cases where posterior approach is performed; fixation of the mesh takes place on the posterior wall of cervix. We utilize nonabsorbable sutures for this step (prolene 3 − 0). The sutures are held in place with Kocher clamps. As a result, a total of four sutures have been placed for the bilateral sacrospinous colposuspension.
7. Threading the sutures through the mesh: All 4 sutures are threaded through the mesh. The median sutures are threaded through the central part of the mesh. The I-Stitch sutures are then threaded through the arms of the mesh. The sutures are now held again with clamps or adhesive tape.
8. Mesh fixation and tying of sutures: The two midline sutures are first tied to fix the central part of the mesh. The I-Stitch sutures are now tied and the knot is guided with the index finger to the fixing point on the sacrospinous ligament. The elevation of the cervix or vaginal apex becomes obvious at this point.
9. Additional colporrhaphy and closure of the vaginal incision: additional sutures for the simultaneous anterior or posterior repair could be placed during this step if needed. Finally, the vaginal wall incision is closed with absorbable running suture.
10. Urinary catheterization and vaginal packing: We routinely insert a Foley catheter and a vaginal gauze coated with estriol creme. The catheter and the gauze are removed on the second postoperative day. This was followed by routine measurement of postvoidal residual volume.

### Statistical analysis

The statistical analysis was carried out using R Software version 3.5.2 (2018-12-20) “Eggshell Igloo”. Data were expressed as mean with standard deviation or frequency and percentage.

Pre- and 12 months postoperative POP-Q results were compared using paired samples Wilcoxon test (Wilcoxon signed-rank test).

The results of preoperative & 12-months postoperative assessment for Prolapse Quality of Life (P-QOL) questionnaire were compared using Fisher’s exact test. The value of p < 0.05 was considered to be statistically significant.

## Results

A total of 47 surgeries were performed during the study period. However, 30 Patients were included in this single center retrospective study. We have only included patients that were able to show up for postoperative evaluation 12 months after the surgery. The demographic data of these women are summerized in Table 1.

**Table 1:** Demographic data of patients (n = 30) at time of BSC operation

Age, y, mean ± SD	64.73 ± 12.65
BMI, kg/m^2^, mean ± SD	27.26 ± 4.38
Menopausal status, n (%)	
Pre-menopausal Post-menopausal	3 (10) 27 (90)
Parity, median (range)	2 (0–9)
ASA, mean (range)	2 (2–3)
Previous hysterectomy, n (%)	
Total hysterectomy Supracervical hysterectomy	9 (30) 2 (7)

BSC: Bilateral Sacrospinal Colposuspension

ASA: American Society of Anesthiologists Physical Status Classification System

The average age of the patients was 64.73 (SD ± 12.65) and the average BMI was 27.26 (SD ± 4.38). 90% of the patients were postmenopausal. The average parity of the patients was 2 (range 0–9) and most patients had an ASA classification 2 (range 2–3). 9 patients (30%) had a history of total hysterectomy and were presenting with vaginal vault prolapse. 2 patients (7%) had a history of supracervical hysterectomy and the rest of patients had an intact uterus at the time of BSC surgery.

Table 2 shows the concomitant procedures that were performed at the time of BSC surgeries. 26 patients (87%) had an anterior colporrhaphy due to an associated cystocele. 4 patients (13%) had a posterior colporrhaphy due to an associated rectocele. Only 2 patients (7%) had a vaginal hysterectomy before the colposuspension due to symptomatic fibroids.

**Table 2:** Concomitant procedures performed at time of BSC operation

Anterior colporrhaphy	26 (87)
Posterior colporrhaphy	4 (13)
Vaginal hysterectomy	2 (7)

Data are given as number (%)

BSC: Bilateral Sacrospinal Colposuspension

No intraoperative adverse events were recorded for all patients. Table 3 summerizes the postoperative complications. There was no record for visceral injury or need for reoperation due to hematoma formation or pain. Urinary tract infections did not occur in any of the patients in the immidiate follow-up period. 2 patients (6.7%) developed postoperative urinary retention. Spontaneous remission occured in both patients after an additional two days of catheterization and medical treatment with Tamsulosin. 3 Patients (10%) complained of postoperative lower back pain and 1 patient (3%) complained of groin pain. The pain was managed conservatively with pain medications and physiotherapy and disappeared after a couple of weeks in all 4 patients. 1 patient (3%) developed a small hematoma that was detected with an ultrasound examination and resolved spontaneously without a surgical intervention. There was no recorded surgery site infection or mesh induced vaginal erosion or visceral injury.

**Table 3:** Postoperative complications

Lower back pain	3 (10)
Groin tendonitis / groin pain	1 (3)
Postoperative hematoma	1 (3)

Data are expressed as number (%)

**Fig. 1:**
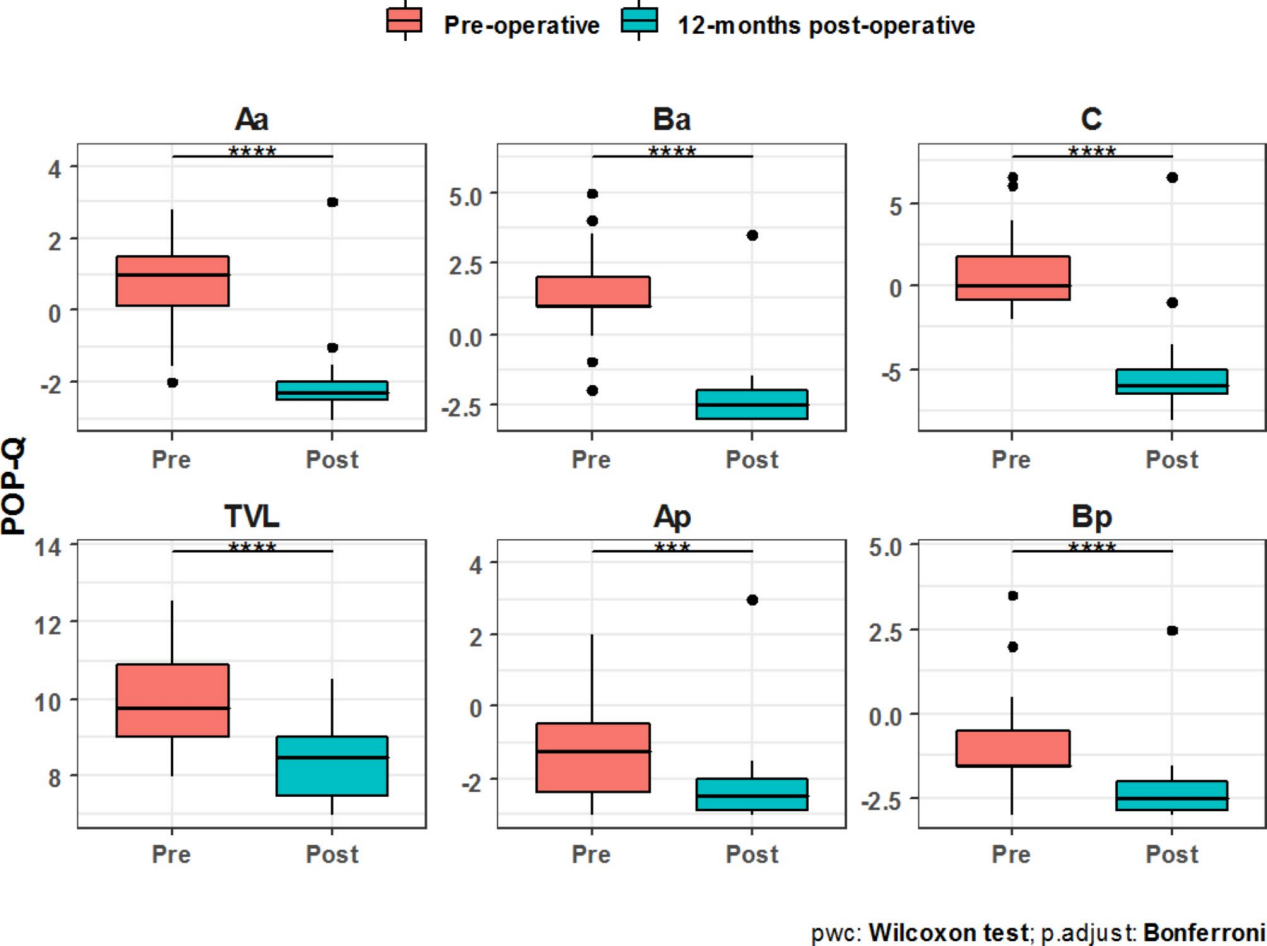
Comparative analysis between pre & postoperative POP-Q assessment

The POP-Q parameters were significantly improved at twelve months after surgery compared to baseline. All women were asymptomatic one year after surgery and had either a stage 0 or stage 1 POP. Total vaginal length was shorter at the one year follow up examination when compared to baseline. (Table 4; Fig. 1)

**Table 4:** Comparative analysis between pre- & 12-months postoperative assessment for pelvic organ prolapse quantification (POP-Q):

POP-Q parameters		Preoperative	12-months postoperative	P value
Aa	**Mean** ± **SD**	0.81 ± 1.21	-2.10 ± 1.08	< 0.001***
Ba	**Mean** ± **SD**	1.37 ± 1.53	-2.27 ± 1.17	< 0.001***
C	**Mean** ± **SD**	0.70 ± 2.25	-5.33 ± 2.59	< 0.001***
TVL	**Mean** ± **SD**	9.98 ± 1.24	8.33 ± 0.90	< 0.001***
Ap	**Mean** ± **SD**	-1.13 ± 1.43	-2.25 ± 1.09	< 0.001***
Bp	**Mean** ± **SD**	-0.83 ± 1.52	-2.27 ± 1.01	< 0.001***

Data are expressed as mean ± standard deviation (SD); Aa: anterior vaginal wall, 3 cm proximal to the hymen; Ba: most distal position of the remaining upper anterior vaginal wall; C: cervix or cuff; TVL: total vaginal length; Ap: posterior vaginal wall, 3 cm proximal to the hymen; Bp: most distal position of the remaining upper posterior vaginal wall.

The Prolapse Quality of Life Questionnaire (P-QoL) is a validated questionnaire created to assess the impact of pelvic organ prolapse on women’s quality of life. We have utilized the validated German version of the questionnaire [21]. The questionnaire has been divided into four domains covering:

1. General health perception, relationships and limitations.
2. Urinary symptoms associated with prolapse.
3. Bowel symptoms associated with prolapse.
4. Other symptoms associated with prolapse.

**Fig. 2:**
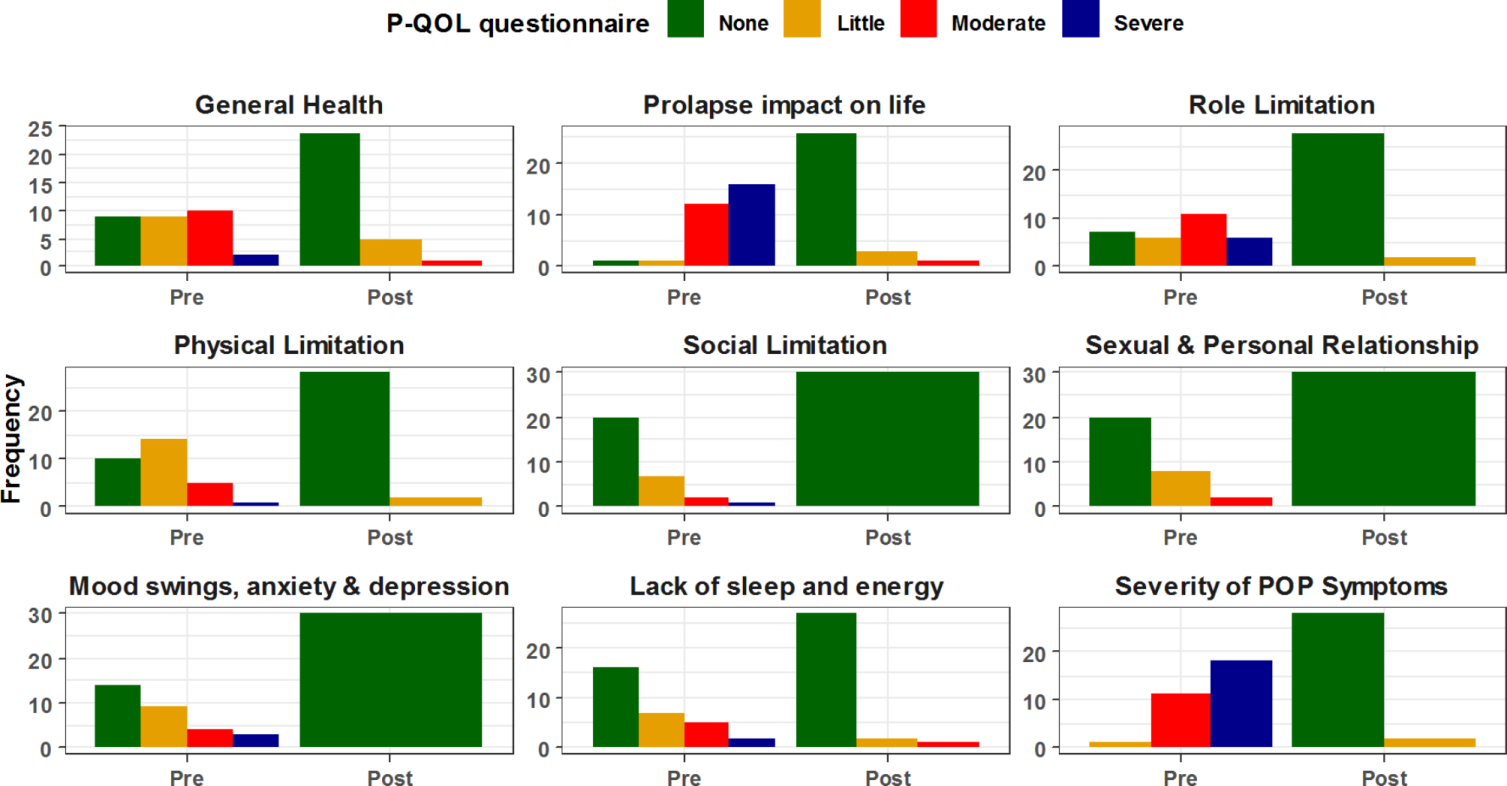
Comparative analysis between pre & postoperative general health assessment

**Fig. 3:**
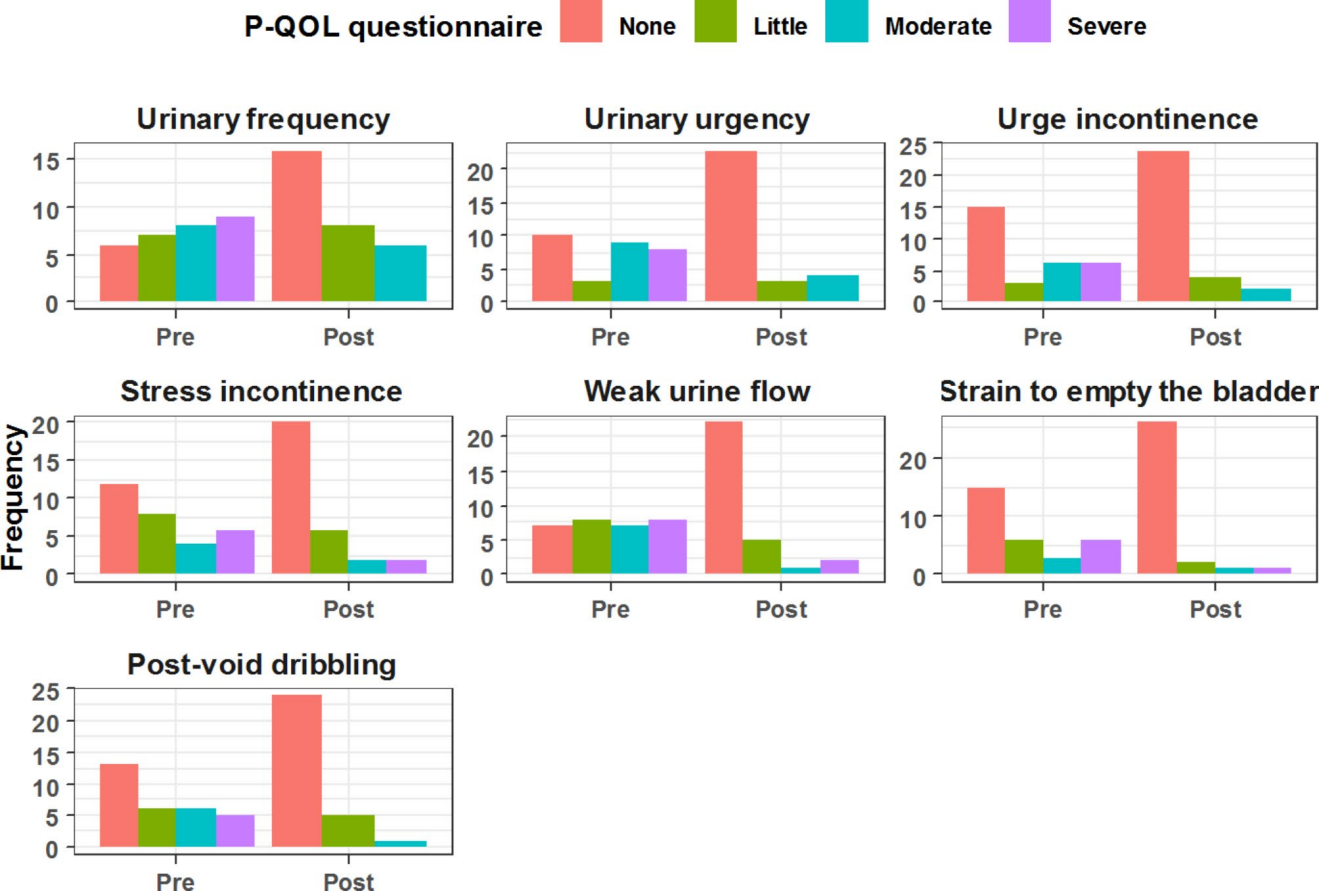
Comparative analysis between pre & postoperative Urinary symptoms assessment

**Fig. 4:**
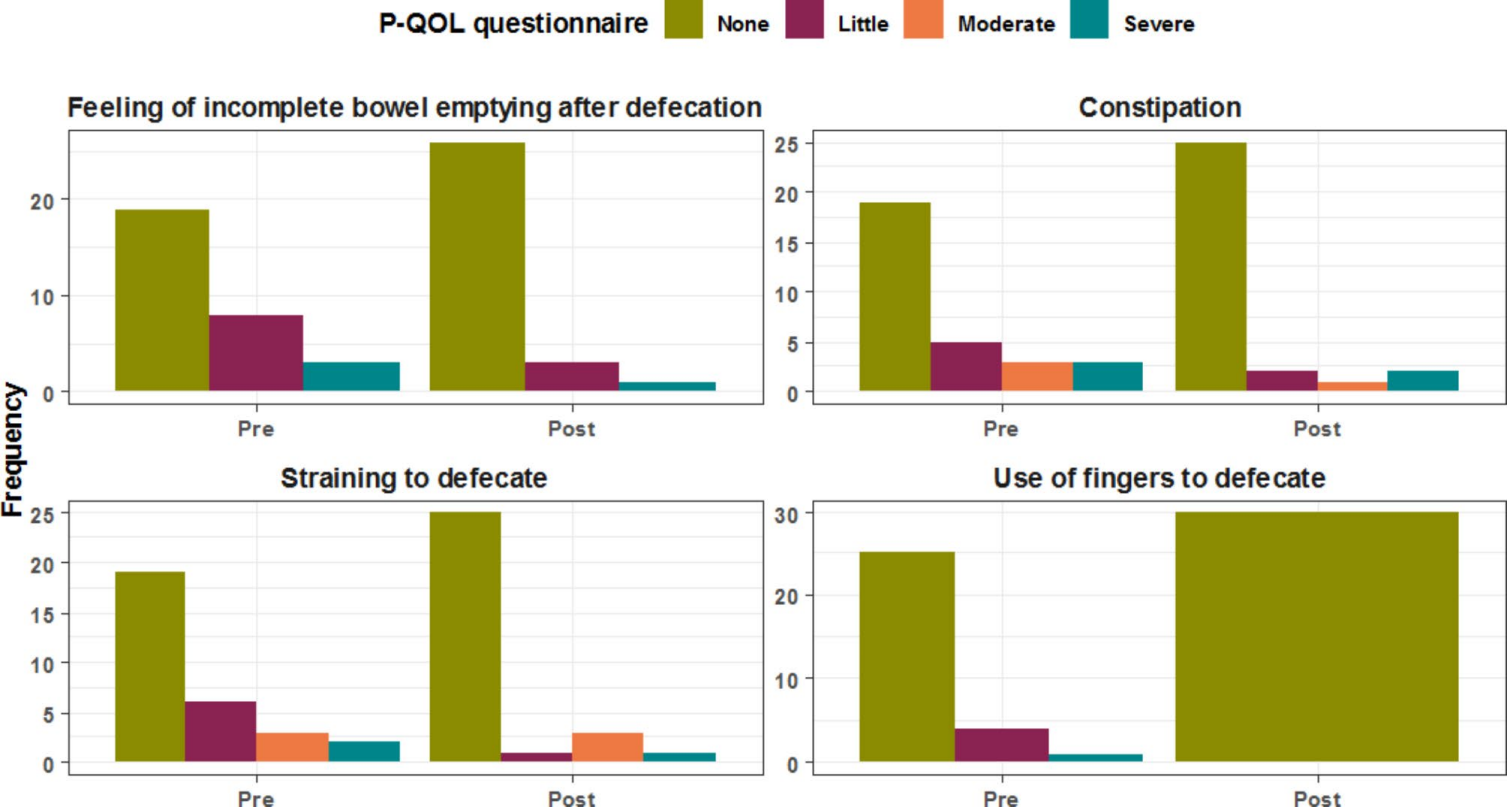
Comparative analysis between pre & postoperative bowel symptoms assessment

**Fig. 5:**
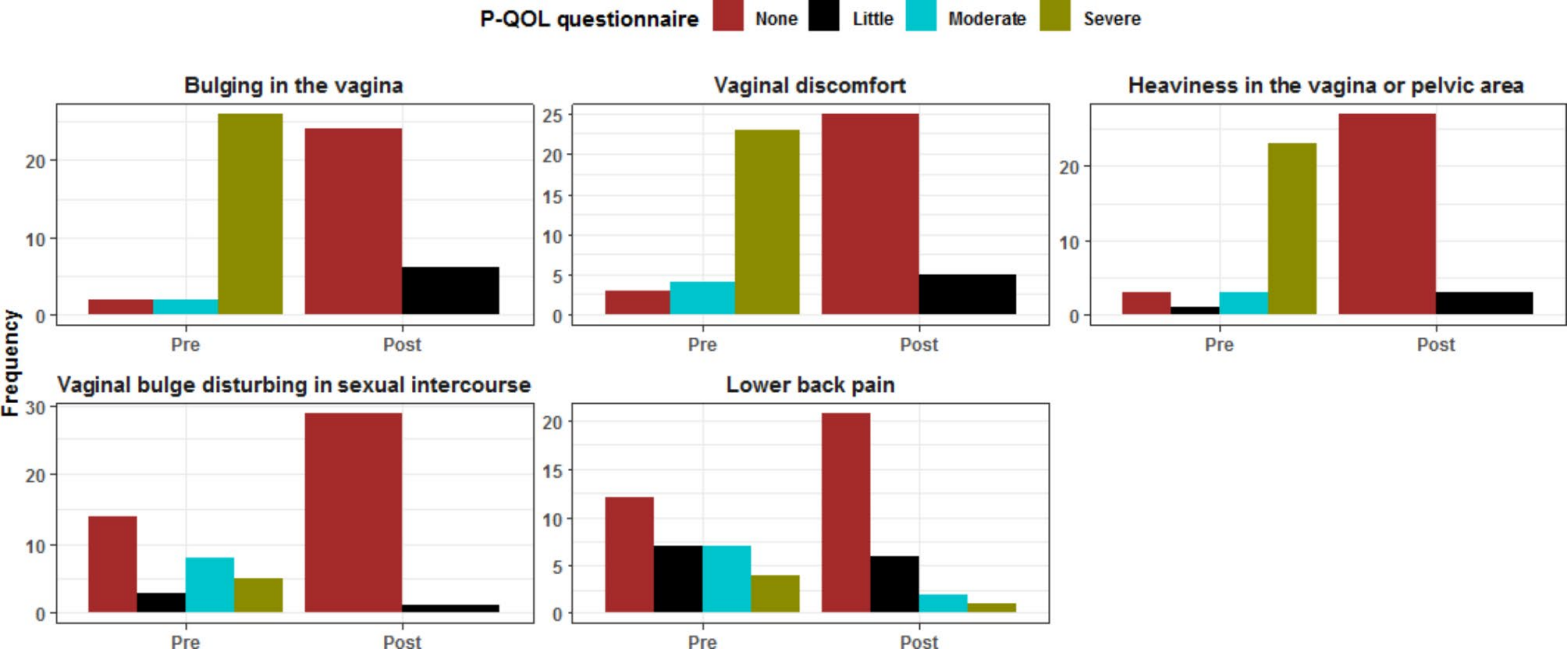
Comparative analysis between pre & postoperative other symptoms assessment

All four subdomains of the P-QOL-questionnaire showed positive trends and improvement at twelve months after surgery when compared to preoperative values. All patients were satisfied with the surgery outcomes at the follow-up time of 12 months. (Tables 5, 6, 7 and 8; Figs. 2, 3, 4 and 5)

**Table 5:** Comparative analysis between pre- & 12-months postoperative assessment for Perceived Quality of Life (P-QOL) questionnaire domains: (General health)

		Preoperative	12-months postoperative	P value
General Health	**None**	9 (30.0%)	24 (80.0%)	< 0.001
	**Little**	9 (30.0%)	5 (16.7%)	
	**Moderate**	10 (33.3%)	1 (3.3%)	
	**Severe**	2 (6.7%)	0 (0.0%)	
Prolapse impact on life	**None**	1 (3.3%)	26 (86.7%)	< 0.001
	**Little**	1 (3.3%)	3 (10.0%)	
	**Moderate**	12 (40.0%)	1 (3.3%)	
	**Severe**	16 (53.3%)	0 (0.0%)	
Role Limitation	**None**	7 (23.3%)	28 (93.3%)	< 0.001
	**Little**	6 (20.0%)	2 (6.7%)	
	**Moderate**	11 (36.7%)	0 (0.0%)	
	**Severe**	6 (20.0%)	0 (0.0%)	
Physical Limitation	**None**	10 (33.3%)	28 (93.3%)	< 0.001
	**Little**	14 (46.7%)	2 (6.7%)	
	**Moderate**	5 (16.7%)	0 (0.0%)	
	**Severe**	1 (3.3%)	0 (0.0%)	
Social Limitation	**None**	20 (66.7%)	30 (100.0%)	0.001
	**Little**	7 (23.3%)	0 (0.0%)	
	**Moderate**	2 (6.7%)	0 (0.0%)	
	**Severe**	1 (3.3%)	0 (0.0%)	
Sexual & Personal Relationship	**None**	20 (66.7%)	30 (100.0%)	0.001
	**Little**	8 (26.7%)	0 (0.0%)	
	**Moderate**	2 (6.7%)	0 (0.0%)	
	**Severe**	0 (0.0%)	0 (0.0%)	
Mood swings, anxiety & depression	**None**	14 (46.7%)	30 (100.0%)	< 0.001
	**Little**	9 (30.0%)	0 (0.0%)	
	**Moderate**	4 (13.3%)	0 (0.0%)	
	**Severe**	3 (10.0%)	0 (0.0%)	
Lack of sleep and energy	**None**	16 (53.3%)	27 (90.0%)	0.011
	**Little**	7 (23.3%)	2 (6.7%)	
	**Moderate**	5 (16.7%)	1 (3.3%)	
	**Severe**	2 (6.7%)	0 (0.0%)	
Severity of POP Symptoms	**None**	0 (0.0%)	28 (93.3%)	< 0.001
	**Little**	1 (3.3%)	2 (6.7%)	
	**Moderate**	11 (36.7%)	0 (0.0%)	
	**Severe**	18 (60.0%)	0 (0.0%)	

Data are represented as frequency & (percentage)

**Table 6:** Comparative analysis between preoperative & 12-months postoperative assessment for Perceived Quality of Life (P-QOL) questionnaire domains: (Urinary symptoms)

Urinary symptoms		Preoperative	12-months postoperative	P value
Urinary frequency	**None**	6 (20.0%)	16 (53.3%)	0.002
	**Little**	7 (23.3%)	8 (26.7%)	
	**Moderate**	8 (26.7%)	6 (20.0%)	
	**Severe**	9 (30.0%)	0 (0.0%)	
Urinary urgency	**None**	10 (33.3%)	23 (76.7%)	0.001
	**Little**	3 (10.0%)	3 (10.0%)	
	**Moderate**	9 (30.0%)	4 (13.3%)	
	**Severe**	8 (26.7%)	0 (0.0%)	
Urge incontinence	**None**	15 (50.0%)	24 (80.0%)	0.013
	**Little**	3 (10.0%)	4 (13.3%)	
	**Moderate**	6 (20.0%)	2 (6.7%)	
	**Severe**	6 (20.0%)	0 (0.0%)	
Stress incontinence	**None**	12 (40.0%)	20 (66.7%)	0.177
	**Little**	8 (26.7%)	6 (20.0%)	
	**Moderate**	4 (13.3%)	2 (6.7%)	
	**Severe**	6 (20.0%)	2 (6.7%)	
Weak urine flow	**None**	7 (23.3%)	22 (73.3%)	0.001
	**Little**	8 (26.7%)	5 (16.7%)	
	**Moderate**	7 (23.3%)	1 (3.3%)	
	**Severe**	8 (26.7%)	2 (6.7%)	
Strain to empty the bladder	**None**	15 (50.0%)	26 (86.7%)	0.02
	**Little**	6 (20.0%)	2 (6.7%)	
	**Moderate**	3 (10.0%)	1 (3.3%)	
	**Severe**	6 (20.0%)	1 (3.3%)	
Post-void dribbling	**None**	13 (43.3%)	24 (80.0%)	0.005
	**Little**	6 (20.0%)	5 (16.7%)	
	**Moderate**	6 (20.0%)	1 (3.3%)	
	**Severe**	5 (16.7%)	0 (0.0%)	

Data are represented as frequency & (percentage)

**Table 7:** Comparative analysis between preoperative & 12-months postoperative assessment for Perceived Quality of Life (P-QOL) questionnaire domains: (Bowel symptoms)

Bowel symptoms		Preoperative	12-months postoperative	P value
Feeling of incomplete bowel emptying after defecation	**None**	19 (63.3%)	26 (86.7%)	0.147
	**Little**	8 (26.7%)	3 (10.0%)	
	**Moderate**	0 (0.0%)	0 (0.0%)	
	**Severe**	3 (10.0%)	1 (3.3%)	
Constipation	**None**	19 (63.3%)	25 (83.3%)	0.344
	**Little**	5 (16.7%)	2 (6.7%)	
	**Moderate**	3 (10.0%)	1 (3.3%)	
	**Severe**	3 (10.0%)	2 (6.7%)	
Straining to defecate	**None**	19 (63.3%)	25 (83.3%)	0.178
	**Little**	6 (20.0%)	1 (3.3%)	
	**Moderate**	3 (10.0%)	3 (10.0%)	
	**Severe**	2 (6.7%)	1 (3.3%)	
Use of fingers to defecate	**None**	25 (83.3%)	30 (100.0%)	0.052
	**Little**	4 (13.3%)	0 (0.0%)	
	**Moderate**	0 (0.0%)	0 (0.0%)	
	**Severe**	1 (3.3%)	0 (0.0%)	

Data are represented as frequency & (percentage)

**Table 8:** Comparative analysis between pre- and 12 months postoperative assessment of Perceived Quality of Life (P-QOL) questionnaire domains: (other symptoms)

Other symptoms		Preoperative	12-months postoperative	P value
Bulging in the vagina	**None**	2 (6.7%)	24 (80.0%)	< 0.001
	**Little**	0 (0.0%)	6 (20.0%)	
	**Moderate**	2 (6.7%)	0 (0.0%)	
	**Severe**	26 (86.7%)	0 (0.0%)	
Vaginal discomfort	**None**	3 (10.0%)	25 (83.3%)	< 0.001
	**Little**	0 (0.0%)	5 (16.7%)	
	**Moderate**	4 (13.3%)	0 (0.0%)	
	**Severe**	23 (76.7%)	0 (0.0%)	
Heaviness in the vagina or pelvic area	**None**	3 (10.0%)	27 (90.0%)	< 0.001
	**Little**	1 (3.3%)	3 (10.0%)	
	**Moderate**	3 (10.0%)	0 (0.0%)	
	**Severe**	23 (76.7%)	0 (0.0%)	
Vaginal bulge disturbing in sexual intercourse	**None**	14 (46.7%)	29 (96.7%)	< 0.001
	**Little**	3 (10.0%)	1 (3.3%)	
	**Moderate**	8 (26.7%)	0 (0.0%)	
	**Severe**	5 (16.7%)	0 (0.0%)	
Lower back pain	**None**	12 (40.0%)	21 (70.0%)	0.074
	**Little**	7 (23.3%)	6 (20.0%)	
	**Moderate**	7 (23.3%)	2 (6.7%)	
	**Severe**	4 (13.3%)	1 (3.3%)	

Data are represented as frequency & (percentage

## Discussion

**Fig. 6:**
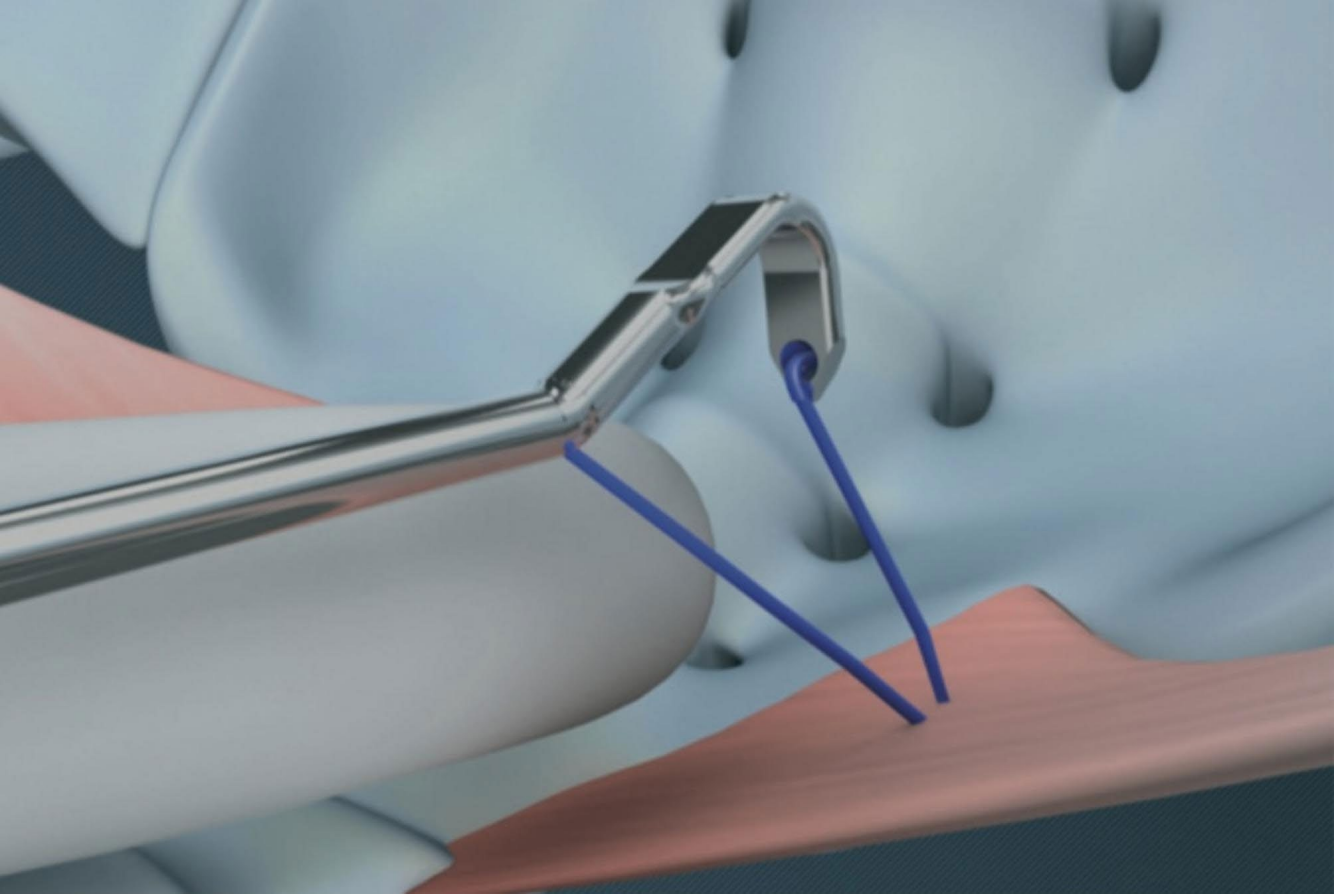
Fixation of the BSC mesh on the medio-cranial aspect of the sacrospinous ligament. Obtained with permission from A.M.I. Austria

The aim of this retrospective study was to assess the anatomical and functional outcomes of the bilateral sacrospinous colposuspension (BSC) in 30 Patients treated with the single incision standardized technique. The utilization of the i-stitch instrument allows minimal invasive access to the fixation points i.e. the medio-cranial aspect of the sacrospinous ligament. (Fig. 6)

**Fig. 7:**
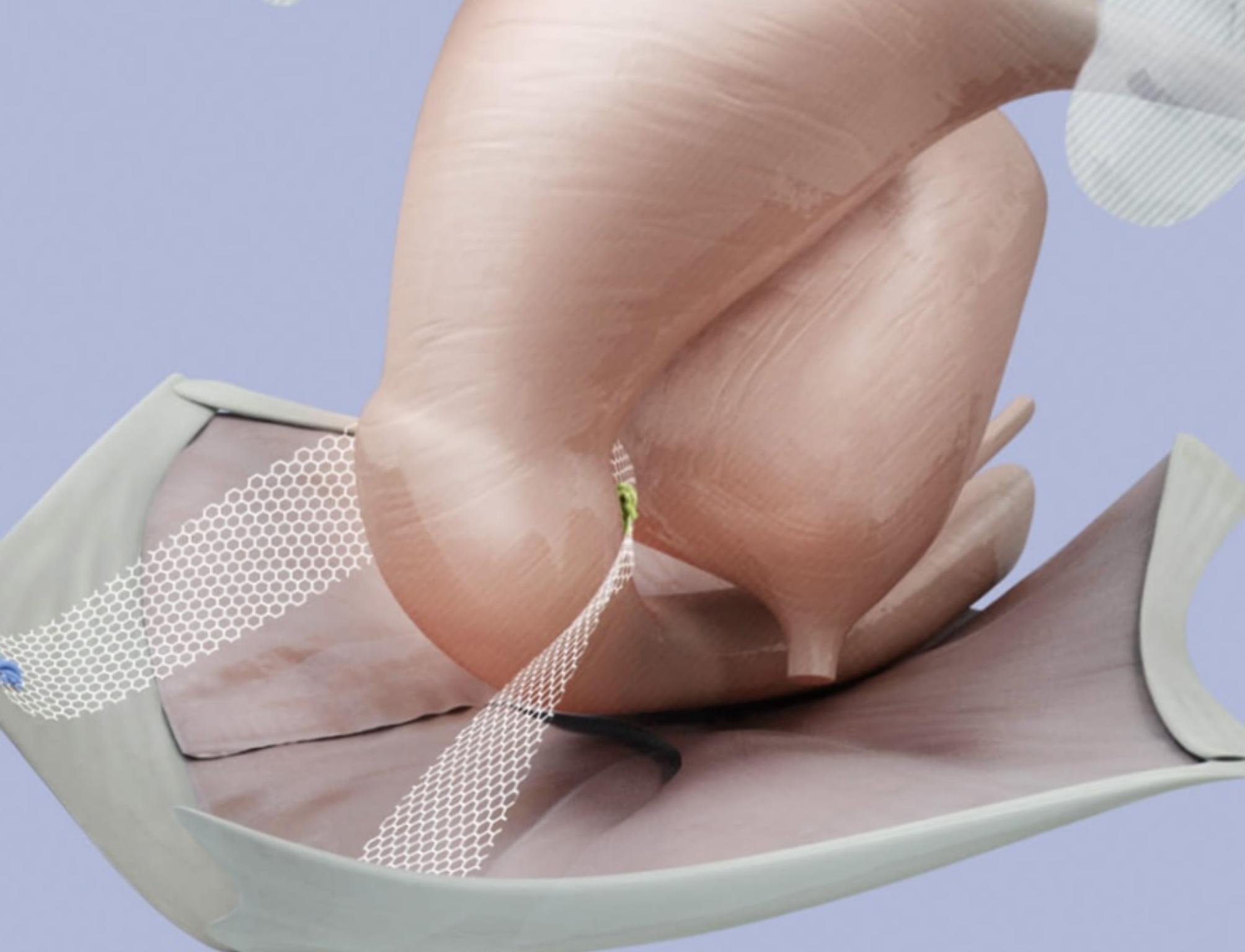
BSC suspending the uterus. Obtained with permission from A.M.I. Austria

The technique offers apical suspension, in the presence or absence of the uterus. The BSC mesh acts like pelvic neo-ligaments and establishes symmetrical, bilateral suspension of the vaginal vault or cervix to the sacrospinous ligaments. This recreates the support previously provided by the natural ligaments which are no longer functioning. (Fig. 7)

Large mesh implants for the management of pelvic organ prolapse are facing scepticism due to possible complications. However, the weight of the BSC-mesh is equivalent to a 0–0 suture and the anchoring technique is minimally invasive.

Till date, very few studies were published evaluating the results of this surgical technique in terms of its functional and anatomical outcomes.

The surgical steps were first described by Kieback in 2019 to allow for the standardization and reproducibility of the technique [20].

In a prospective study of 132 patients with vaginal prolapse; surgical and functional outcomes of BSC have been evaluated with 6 months follow-up. The authors concluded that BSC is an efficient minimal inavasive technique for the treatment of female apical prolapse with a very favourable risk / benefit ratio [22].

Another study evaluated the quality of life after surgical treatment of isolated apical defects with BSC. The study involved 60 patients and was more focused on the assessment of patients` satisfaction after the surgery. The authors concluded that in most patients, surgical treatment of isolated apical defects using BSC-mesh resulted in the elimination of bothersome symptoms and improvement of quality of life [23].

A thorough literature research revealed no other published studies assessing the outcomes of BSC as a minimally invasive vaginal surgery for the treatment of apical prolapse.

The results of our study show that bilateral sacrospinous colposuspension is reproducible, minimal invasive, and results in high patient´s satisfaction. The POP-Q data demonstrate that BSC establisches adequate pelvic support for genital organ prolapse. The comparison of our preoperative and postoperative POP-Q results revealed strong significant differences for points Aa, Ba, C, Ap, and Bp (P < 0.001). The bilateral fixation of the mesh on the sacrospinous ligaments ensures that the vaginal axis is close to the original anatomic position. Unilateral sacrospinous fixation may be satisfactory, however, it is associated with deviation of the vaginal axis and may lead to dysparunia and altered bowel functions [24].

The study revealed that in addition to significantly improving patients‘ quality of life, urogenital symptoms and also bowel symptoms have improved postoperatively. A good argument here is that this improvement is a result of the concomitant anterior or posterior colporrhaphy. However, patients with pelvic organ prolapse experience symptoms that do not necessarily correlate with compartment-related defects [25]. Due to this poor correlation between the prolapsed compartments and symptomatology; it is difficult in combination prolapse surgery to attribute the entire improvement of symptoms to a certain procedure.

As described earlier; most complications were self limiting. There were no intra- or postoperative complications that required a surgical intervention or removal of mesh. Other complications that have been associated with vaginal mesh are mesh exposure and dysparunia. None of the 30 patients had complained of dysparunia or had a mesh exposure in the 12 months postoperative period. This outcome maybe linked to several factors including routine pre- and postoperative local vaginal treatment with estrogen, avoidance of extensive tissue dissection or manipulation, avoidance of concomitant sling procedures for SUI, the type of mesh used, and fixation of the mesh in a tension-free manner.

The surgeries were conducted in general anaesthesia as we wanted to follow the exact same surgical steps previously published. This allowed us to ensure that the procedure is standardized and reproducible. The authors believe that the surgery can probably still be performed rapidly and safely in regional anesthesia or using local anesthesia with sedation.

Obviously, there are limitations to our study. This includes the relatively small sample size and the retrospective nature of the study. The follow-up time of 12 months is considered short and long-term results of the procedure should be evaluated. All procedures were performed by the same two experienced surgeons, hence a prospective multicenter study with a longer follow up duration would be of great value in validating the excellent outcomes of this study.

The FDA has imposed restrictions to the transvaginal use of mesh as treatment option for POP. There has been increased reports of transvaginal mesh related complications. The current guidline of the German Working Group of the Scientific Medical Societies (AWMF), the European Urogynecological Association (EUGA) and the European Association of Urology (EAU) permit the differentiated and regulated use of synthetic transvaginal meshes in POP repair. It was highlighted in the “German-Austrian-Swiss Guidline for Management of POP” that transvaginal mesh implants are superior to native tissue repair in terms of objective success rates and recurrence rates [26].

Given the FDA warnings on vaginal mesh, conventional transvaginal native tissue repair with increased recurrence rates are rising. Additionally, more invasive and longer abdominal and laparoscopic approaches have been increasingly utilized for the management of POP.

The use of an ultralight weight mesh product for the minimally invasive transvaginal management of POP is an interesting topic that warrants more research and long-term follow up for patients. The authors believe that it is crucial that patients are assessed and treated individually. Clear indications, standardized procedures, surgical expertise, shared decision making with patients, high quality ultralight mesh and more research are important for acheiving the best possible outcomes for our patients.

## Conclusion

This study highlights the functional and anatomical outcomes of the minimally invasive vaginal bilateral sacrospinal colposuspension with ultralight mesh for the management of apical prolapse. The one year postoperative results of the proposed procedure reflect excellent outcomes with minimal complications. The data published here are very promising and warrant further investigations and more studies to evaluate the long-term outcomes of BSC in the surgical management of apical defects.

## Data Availability

The datasets used and analysed during the current study are available from the corresponding author on reasonable request.

## Abbreviations

BSC: Bilateral sacrospinal colposuspension
POP-Q: Pelvic organ prolapse quantification system
P-QOL: Prolapse quality of life
POP: Pelvic organ prolapse
SUI: Stress urinary incontinence
BMI: Body mass index.
